# Supplementary material for: Plasma Heating Induced by Tadpole-like Downflows in the Flaring Solar Corona
Source: Innovation (Camb). 2021 Jan 19;2(1):100083. doi: 10.1016/j.xinn.2021.100083 (PMC8454670; doi:10.1016/j.xinn.2021.100083)
Supplement: Figure S1. Results of Wavelet Analysis for the Time Series of the AIA 131 Å Intensity and DEM Temperature Averaged over the Whole Region of Figure 6A — (A) Top panel shows the normalized intensity variation of AIA 131 Å. The bottom left panel shows the wavelet power spectrum. The cross-hatched region above the wavelet power spectrum highlights the cone of influence (COI). The location of power above 99.99% significance level is represented by the region overplotted with dotted white lines. The bottom right panel shows the global wavelet power. The longest measurable period is 11.1 minutes (due to the COI), which is indicated by a horizontal dashed line. The dotted line shows the significance level of 99.99%. The significant periods that are measured from the global wavelet power are printed at the top of the global wavelet power plot. (B) The panels are the same as in (A) but for the DEM temperature. [file mmc1.pdf]

The Innovation, Volume *2*

Supplemental Information

Plasma Heating Induced by Tadpole-like Downflows in the Flaring Solar Corona

Tanmoy Samanta, Hui Tian, Bin Chen, Katharine K. Reeves, Mark C.M. Cheung, Angelos Vourlidas, and Dipankar Banerjee

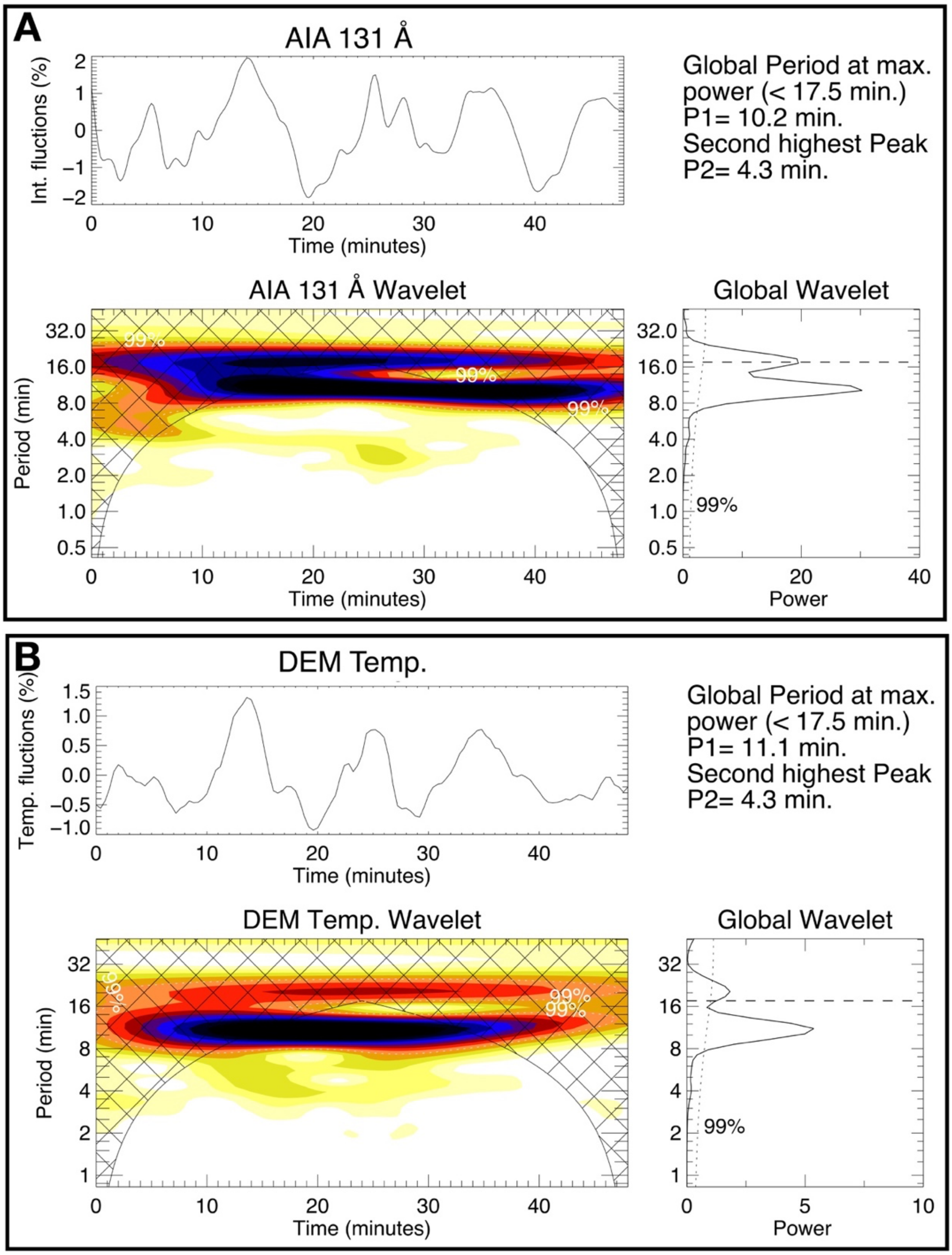

Figure S1: Results of wavelet analysis for the time series of the AIA 131 Å intensity and DEM temperature averaged over the whole region of Figure 6A. (A) Top panel shows the normalized intensity variation of AIA 131 Å. The bottom left panel shows the wavelet power spectrum. The cross-hatched region above the wavelet power spectrum highlights the cone of influence (COI). The location of power above 99.99 % significance level is represented by the region overplotted with dotted white lines. The bottom right panel shows the global wavelet power. The longest measurable period is 11.1 minutes (due to the COI), which is indicated by a horizontal dashed line. The dotted line shows the significance level of 99.99 %. The significant periods that are measured from the global wavelet power are printed at the top of the global wavelet power plot. (B) The panels are the same as in (A) but for the DEM temperature.
